# Enhanced degradation of doxycycline by citric acid-functionalized graphitic carbon nitride decorated with MIL-88A and FeS: optimization, degradation mechanism, and degradation pathway

**DOI:** 10.1039/d5ra07120h

**Published:** 2025-12-10

**Authors:** Abdelazeem S. Eltaweil, Mohammed Salah Ayoup, Jawaher Y. Al Nawah, Eman M. Abd El-Monaem

**Affiliations:** a Department of Engineering, Faculty of Technology and Engineering, University of Technology and Applied Sciences Ibra Sultanate of Oman; b Chemistry Department, Faculty of Science, Alexandria University Alexandria Egypt; c Department of Chemistry, College of Science, King Faisal University Al-Ahsa 31982 Saudi Arabia jalnawah@kfu.edu.sa; d Advanced Technology Innovation Borg El-Arab Alexandria Egypt emanabdelmonaem5925@yahoo.com

## Abstract

This investigation provides a new Fenton-like heterogeneous catalyst construct, citric acid-functionalized graphitic carbon nitride decorated with MIL-88A and iron sulfide (FeS/MIL-88A@Cit–gCN). The characteristics of FeS_0.5_/MIL-88A_0.5_@Cit–gCN were scrutinized using different instruments to identify its surface charge, morphology, elemental and structural compositions, and crystallinity. The catalytic activity of FeS_0.5_/MIL-88A_0.5_@Cit–gCN was inspected by a series of adsorption/Fenton-like experiments, evaluating the best catalytic parameters for efficiently decomposing doxycycline (Dox). The maximum adsorption% and decomposition% of Dox were 48.78% and 99.40%, respectively, at H_2_O_2_ concentration = 100 mg L^−1^, system temperature = 20 °C, pH = 5, and FeS_0.5_/MIL-88A_0.5_@Cit–gCN dose = 0.01 g. The second-order kinetic model best represented the Dox decomposition process by FeS_0.5_/MIL-88A_0.5_@Cit–gCN. The decomposition mechanism of Dox proceeded by a catalytic radical pathway, and most probably, ˙OH was the governing radical in the catalytic medium. The ˙OH radicals were produced through the contribution of the iron, sulfur, and electron-donor groups of FeS_0.5_/MIL-88A_0.5_@Cit–gCN to activate H_2_O_2_. The adsorption reaction played an excellent role in the decomposition capacity of Dox since the drug molecules were attached to the FeS_0.5_/MIL-88A_0.5_@Cit–gCN surface by n–pi interactions, coulombic interactions, and coordination bonds. The recycling study denoted the durability of FeS_0.5_/MIL-88A_0.5_@Cit–gCN after reusing for five times. These results render FeS_0.5_/MIL-88A_0.5_@Cit–gCN a premium heterogeneous catalyst that can be applied at an industrial scale.

## Abstract 2

The FeS_0.5_/MIL-88A_0.5_@Cit–gCN catalyst was fabricated to efficiently degrade doxycycline *via* Fenton-like oxidation, with excellent stability and reusability.

## Graphical abstract

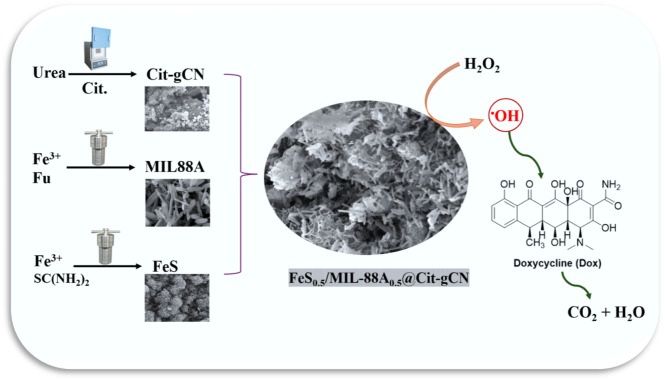

## 1. Introduction

Drinking water shortage is an apprehending global concern due to the prevalence of pollution in most water resources. One of the biggest sources of water pollution is antibiotic residues, such as doxycycline (Dox), which is an exceptional anti-inflammatory and anti-apoptotic drug suitable for treating the current pandemic diseases.^1,2^ Large Dox quantities are used daily for curing infections, such as those in the stomach, skin, urinary tract, and respiratory tract. Meanwhile, about half of the Dox dosages cannot be digested by the human body and are excreted *via* urine.^3^ For this sake, environmental researchers are engaged in ameliorating the efficacy of wastewater remediation processes. Amongst them, the Fenton process is an advanced hydroxyl radical (˙OH)-based oxidation reaction that involves decomposing toxic organic contaminants into eco-friendly compounds.^4,5^ The Fenton reaction proceeds through the activation of hydrogen peroxide (H_2_O_2_) by iron catalysts to produce ˙OH, which is responsible for attacking the targeted organic pollutants in the catalytic medium.^6^ Nevertheless, the catalytic activity of iron catalysts and their redox Fe^2+^/Fe^3+^ cycle still needs further improvement.^7^ Notably, several studies have recommended the remarkable catalytic efficiency of non-iron (Fenton-like) catalysts, including layered double hydroxides, metal oxides/sulfides, carbon materials, and metal–organic frameworks.^8^

Graphitic carbon nitride (gCN) is a layer-shaped polymer that is prepared *via* pyrolyzing nitrogen-containing substances, such as dicyandiamide, cyanamide, melamine, guanidine hydrochloride, and urea.^9^ Pioneering studies have suggested that urea-derived gCN could be a better choice than other nitrogen-containing substances, as urea is an eco-friendly and low-cost substance, which makes it appropriate for large-scale applications.^10^ In addition, the self-supported gas preparation approach using urea as a precursor is distinguished from other template synthetic methods, in which urea produces gas bubbles during thermal treatment that act as a soft template and facilitate the formation of porous microstructures.^11^ Furthermore, this produced gas has another significant role in the processing carbon nitride condensation.^12^ Notably, by comparing the soft-template approach to the other template approaches, it was deduced that the soft-template approach has simple processing, does not form impurities, and is suitable to yield a porous microstructure.

The structure of gCN is composed of arranged tri-*s*-triazine units, each comprising six nitrogen atoms.^13^ The gCN possesses several interesting merits, including high chemical stability, low costs, eco-benign properties, a high surface area, facile accessibility, and excellent catalytic activity.^14^ The individual structure of gCN contains many lone pairs of electrons, making it an outstanding Fenton-like catalyst. Moreover, the structural flexibility of gCN eases fostering its catalytic Fenton-like activity by forming a composite or functionality. Citric acid (Cit) serves as a potent chelating agent and exhibits remarkable Fenton-like catalytic activity, making it suitable for the functionalization of gCN. Citric acid is a short-chain carboxylic acid that contains abundant carboxyl and hydroxyl groups.^15,16^ Surprisingly, citric acid has demonstrated excellent performance in the Fenton-like degradation of organic pollutants, as it can activate H_2_O_2_ by sharing its ample electrons. Additionally, citric acid is a chelating agent; so, it can easily chelate contaminants on its surface.^17^ Notably, citric acid possesses a significant chelation property towards iron species, which prevents iron precipitation at higher pH values.^18^

Several published papers have documented the good Fenton-like catalytic activity of gCN for degrading pharmaceutical drugs. In this context, Haroon *et al.*^19^ fabricated Fe_3_O_4_-doped gCN to degrade TC *via* a Fenton-like reaction. The maximum decomposition% of TC by Fe_3_O_4_-doped gCN was 90.00% when the doped Fe_3_O_4_ percentage was 20%. The magnetic character enhanced the recyclability of Fe_3_O_4_-doped gCN, and the recycling test elucidated a slight decline in its activity after reuse in the decomposition reaction of TC. Furthermore, Mu *et al.*^20^ studied the catalytic activity of magnetic Fe-gCN for Fenton-like degradation of APAP, implying that the decomposition% was nearly 100% after 30 minutes over a wide pH scale. In another study, Liu and his co-authors^21^ focused on photo-Fenton degradation of Dox by gCN@MIL-100 in the persulfate and H_2_O_2_ systems. The degradation percentages of Dox by activating persulfate and H_2_O_2_ using gCN@MIL-100 were 82.80% and 69.20% after 30 minutes, respectively. This result was attributed to the generation of higher active radicals by persulfate than by H_2_O_2_.

In the recent decade, bountiful MOF families have been deemed superb candidates for applications in various sectors, including gas separation, solar cells, drug delivery, catalysis, sensors, and others.^22,23^ MOFs possess discernible properties, including a high surface area, excellent mechanical and thermal behavior, a porous structure, and easy functionalization. Noteworthily, the chemical structures of MOFs render them remarkable Fenton/Fenton-like catalysts because they comprise unsaturated metal ions and active oxygen groups.^24^ Because of the auspicious catalytic activity, excellent chemical structure, and reusability of MIL-88A, it exhibited promising results when it was applied in the Fenton degradation reactions of many toxic organic pollutants.^25^ Based on the synthesis approach of MIL-88A, it is classified as a hexagonal MIL-88A when prepared by the solvothermal method using *N*,*N*-dimethylformamide as the reaction solvent. Similarly, the rod-shaped MIL-88A is fabricated *via* the hydrothermal method in an aqueous medium, which is a favorable preparation process owing to the usage of an eco-friendly solvent.^26,27^

Diverse investigations have reported the high catalytic activity of MIL-88A toward pharmaceutical drugs; for instance, Shi *et al.*^28^ decorated MIL-88A with MgFe_2_O_4_ to design a magnetic catalyst by a ball-milling method. The MgFe_2_O_4_/MIL-88A catalyst showed remarkable photo-Fenton catalytic activity for degrading SMX, in which the decomposition percentage reached 99.80% after 20 minutes. Furthermore, Wang and his co-workers^29^ engineered the photo-Fenton MIL-88A/cotton fiber catalyst for decomposing tetracyclines. The degradation percentages of OTC, TC, and CTC by activating PDS using MIL-88A/cotton fibers in the presence of UV light were 97.5%, 95.2%, and 100.0%, respectively. In this context, Eltaweil *et al.*^30^ synthesized Ce-decorated MIL-88A/gCN to decompose TC *via* the oxidative Fenton-like reaction. The experimental results demonstrated a synergistic effect between adsorption and Fenton-like decomposition reactions, where the adsorption% and decomposition% of TC were 51.96% and 92.44%, respectively, in a neutral pH medium. Although MIL-88A has been applied in the photo-Fenton/Fenton degradation of many pharmaceutical drugs, it has not yet been applied in the Fenton decomposition of Dox.

Metal sulfides are materials built *via* a coordination bond between an earth-rich metal (*viz.*, strontium, iron, copper, tin, and nickel) and sulfur species.^31^ Metal sulfides can be prepared *via* many approaches, such as thermal decomposition, precipitation, ball milling, and hydrothermal and electrochemical approaches.^32^ Metal sulfides have shown remarkable outcomes in various fields, including catalysis, CO_2_ reduction, supercapacitors, oxygen generation reactions, and lithium-ion batteries.^4,5^ Furthermore, the prime catalytic activity of metal sulfides in Fenton/Fenton-like processes is due to their being electron-rich nature with good chemical stability and electric conductivity.^33^ The sulfur species of metal sulfides boost the electron transfer from unsaturated metal ions to H_2_O_2_ molecules, which increases the concentration of the yielded ˙OH.^34,35^

Despite the outstanding catalytic performance of metal sulfides, there is a lack of published research that investigates the Fenton/Fenton-like degradation of toxic pharmaceutical drugs by metal sulfides. In one attempt, Yang *et al.*^36^ highlighted the decomposition of OTC by H_2_O_2_ activation using the RS-FeS and RSBC-FeS catalysts. The decomposition% of OTC by RS-FeS and RSBC-FeS were 70.14% and 79.35%, while their adsorption capacities were 635.66 and 827.80 mg g^−1^, respectively. The mechanistic study demonstrated the contribution of both radical and non-radical degradation pathways in decomposing OTC by RS-FeS and RSBC-FeS. In another investigation, Cai *et al.*^37^ studied the Fenton decomposition activity of FeS towards the TC drug, and the decomposition% achieved was 98.00% within 30 minutes at pH = 3. The selectivity test revealed the higher selectivity for TC than that for TOC, with a decomposition% of 20.00% after 60 minutes.

Based on the literature review on the Fenton-like degradation activities of gCN, MIL-88A, and FeS, there is a scarcity of research papers that involve decomposing Dox. The conducted studies on decomposing Dox by gCN, MIL-88A, and FeS have applied the photo-Fenton reaction, but the Fenton degradation mechanism and degradation pathway in the dark have not been investigated. Accordingly, our research focused on boosting the Fenton-like catalytic activity of gCN by the Cit functionality, followed by binding Cit–gCN with iron-based catalysts like MIL-88A and FeS to yield the heterogeneous FeS/MIL-88A@Cit–gCN catalyst. Various characterization instruments were used for investigating the well-fabrication of FeS/MIL-88A@Cit–gCN and its chemical/physical properties. The optimization of the Fenton-like catalytic degradation of Dox by the FeS/MIL-88A@Cit–gCN catalyst was conducted through a series of adsorption/Fenton-like experiments under varied reaction conditions. The resultant experimental data were analyzed by first-order and second-order kinetics. The catalytic mechanism of Dox degradation by FeS/MIL-88A@Cit–gCN was determined using the scavenger test and the XPS spectra of the used and pristine catalysts. The recyclability of the FeS/MIL-88A@Cit–gCN catalyst was determined by performing the cycling test for six adsorption/Fenton-like runs for the Dox molecules.

## 2. Experimental work

### 2.1. Fabrication of Cit–gCN

First, gCN was prepared by the pyrolysis approach as follows: 5.0 g of urea was added to a sealed porcelain crucible and calcined at 550 °C for four hours in a muffle furnace at a heating rate of about 2 °C per minute. The crucible was cooled naturally, and then, the formed gCN was stored for later use. Second, 1 g of gCN was dispersed in 25 mL of distilled water under sonication for 15 minutes. 0.3 M Cit was added to the gCN suspension, followed by sonication for another 15 minutes. Then, the Cit–gCN suspension was kept overnight under slow stirring, allowing Cit and gCN to link together. The Cit-functionalized gCN was separated, rinsed to eliminate excess Cit, and dried at 60 °C in an air oven for 10 hours.

### 2.2. Fabrication of MIL-88A

1.4 g of FeCl_3_·6H_2_O and 0.58 g of fumaric acid were added to 60 mL of distilled water under high-rate stirring. After an hour, the light-orange solution of Fe^3+^/fumaric acid was added to the autoclave, followed by heating it at 85 °C for 24 hours. After cooling the autoclave in the air, the MIL-88A powder was collected using a centrifuge and washed with distilled water and ethanol. Ultimately, the wet MIL-88A powder was kept in an oven at 85 °C for drying.

### 2.3. The fabrication of FeS

2.00 g of FeCl_3_·6H_2_O and 1.15 g of SC(NH_2_)_2_ were added to 80 mL of a mixed solvent of H_2_O and EG with a volume ratio of 3 : 1. The reaction solution was transferred into the autoclave and placed in an oven for heating at 190 °C for 6 hours. The formed black powder was centrifuged, rinsed with distilled water, and heated for 10 hours at 60 °C in an oven.^38^

### 2.4. The fabrication of FeS/MIL-88A@Cit–gCN

In a container, Cit–gCN was suspended in 30 mL of distilled water using an ultrasonic water bath for 30 minutes. Next, FeS and MIL-88A were added to the Cit–gCN suspension, followed by sonication for another 15 minutes. The FeS/MIL-88A@Cit–gCN composite was centrifuged and dried at 70 °C for 10 hours. Three composites were fabricated with different mass ratios of FeS, MIL-88A, and Cit–gCN, named FeS_0.5_/MIL-88A_0.5_@Cit–gCN (0.5 : 0.5 : 1), FeS_0.5_/MIL-88A@Cit–gCN_0.5_ (0.5 : 1:0.5), and FeS/MIL-88A_0.5_@Cit–gCN_0.5_ (1 : 0.5 : 0.5).

### 2.5. Fenton-like degradation experiments of Dox

The catalytic parameters of the Fenton-like decomposition process of Dox by the heterogeneous FeS/MIL-88A@Cit–gCN catalyst were optimized as follows: (1) the decomposition efficacies of Dox by FeS, MIL-88A, Cit–gCN, and FeS/MIL-88A@Cit–gCN composites were investigated to evidence the significance of the binding of the pure components in a composite form and to choose the best mass ratio of these components. (2) The appropriate pH medium for decomposing the Dox molecules efficiently was recorded after comparing the decomposition% of Dox at varied pH values of 3, 5, 7, 9, and 11. (3) The suitable concentration of H_2_O_2_ to decompose the Dox molecules was selected based on the experimental results of decomposing Dox by FeS/MIL-88A@Cit–gCN in the presence of H_2_O_2_ at concentrations of 10, 50, 100, and 200 mg L^−1^ (4) The economical FeS/MIL-88A@Cit–gCN dose to decompose Dox with higher efficiency was determined by studying the process at different dosages of 5, 7.5, 10, and 15 mg. (5) The impact of the system temperature of Dox-FeS/MIL-88A@Cit–gCN was deduced at the temperatures of 20, 30, 40, and 50 °C. (6) The decomposition efficiency of FeS/MIL-88A@Cit–gCN for various Dox concentrations was identified in a concentration range from 50 to 300 mg L^−1^. (7) The recycling test of the FeS/MIL-88A@Cit–gCN catalyst was conducted for six runs of the Dox adsorption/Fenton-like process by separating the catalyst after each run, washing it with an aqueous NaOH solution, and finally drying the composite before using it in the subsequent run. (8) The scavenging test of decomposing Dox by FeS/MIL-88A@Cit–gCN was executed in the presence of TCM and *t*-BuOH as quenchers for O_2_^˙−^ and ˙OH, respectively.

After each experiment, the Dox solution was withdrawn to measure its concentration using a spectrophotometer (labeled as *C*_*t*_) and taking into consideration the concentration of Dox before the degradation reaction (labeled as *C*_0_) to calculate the decomposition% of Dox by eqn (1).1
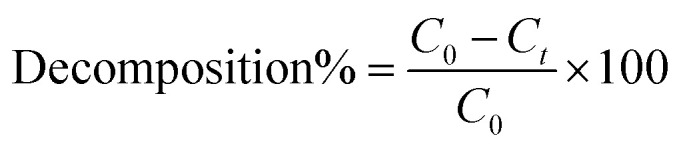


## 3. Results and discussion

### 3.1. Characterization of FeS_0.5_/MIL-88A_0.5_@Cit–gCN

#### 3.1.1. FTIR

**Fig. 1:**
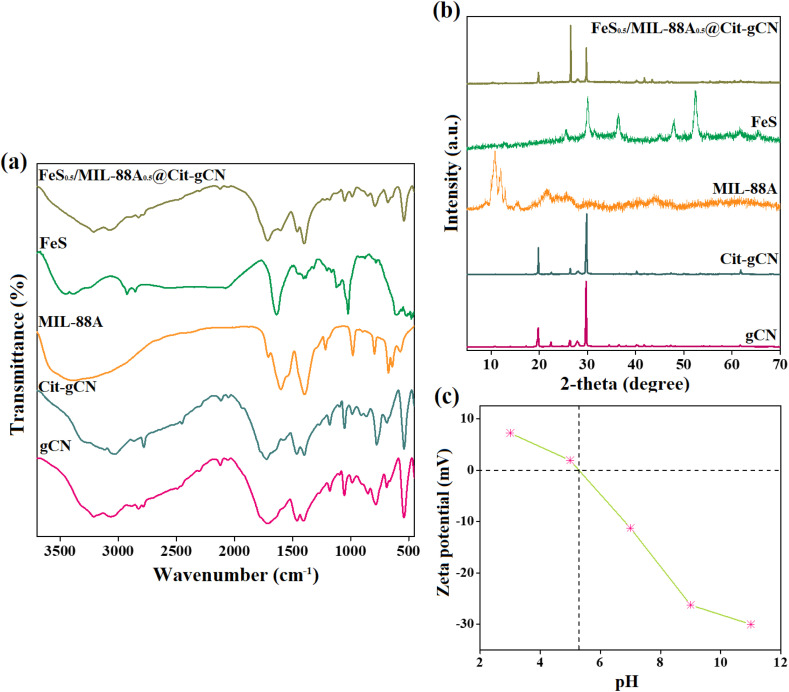
(a) FTIR spectra of FeS, MIL-88A, gCN, Cit–gCN, and FeS_0.5_/MIL-88A_0.5_@Cit–gCN, (b) XRD patterns of FeS, MIL-88A, gCN, Cit–gCN, and FeS_0.5_/MIL-88A_0.5_@Cit–gCN, and (c) ZP of FeS_0.5_/MIL-88A_0.5_@Cit–gCN.

The compositions of FeS, MIL-88A, gCN, Cit–gCN, and FeS_0.5_/MIL-88A_0.5_@Cit–gCN were investigated by the FTIR instrument, as signalized in Fig. 1a. The gCN spectrum elucidated the peaks belonging to primary and secondary amines at 3212 and 3062 cm^−1^, respectively. The absorption peaks of C=N and C–N were observed at 1722 and 1177 cm^−1^, respectively, while the peak related to the gCN heptazine ring appeared at 782 cm^−1^. In the Cit–gCN spectrum, the peaks corresponding to C–O and C=O were revealed at 1253 and 1580 cm^−1^, respectively, indicating the Cit functionality on the gCN surface. In the MIL-88A spectrum, the peak corresponding to the Fe–O bond was manifested at 552 cm^−1^, confirming the linkage of the Fe^3+^ species of the oxygenated groups of fumaric acid. Furthermore, the peaks of C–H and C–C were observed at 1212 and 982 cm^−1^, respectively, which are assigned to the *trans*-diene of the -fumaric acid. In addition, the carboxyl group peaks of the fumaric acid appeared at 1551 and 1398 cm^−1^, which were ascribed to asymmetric and symmetric carboxyl vibrations, respectively. The broad peak centered at around 3400 cm^−1^ was attributed to the hydroxyl of the adsorbed H_2_O molecules.^23–25^ In the FeS spectrum, the absorption peak at 1453 cm^−1^ was related to the C–N bond of SC(NH_2_)_2_, and the peaks at 463, 3437, and 1022 cm^−1^ were attributed to Fe–S, O–H, and Fe–OH, respectively. The peaks of the S–S bond emerged at 616 and 1636 cm^−1^, while the S–O peak appeared at 1117 cm^−1^.^39–41^ In the FeS_0.5_/MIL-88A_0.5_@Cit–gCN spectrum, the peaks belonging to FeS, MIL-88A, and Cit–gCN were observed, confirming their successful linkage inside the catalyst's matrix.

#### 3.1.2. XRD

The crystallographic nature of FeS, MIL-88A, gCN, Cit–gCN, and FeS_0.5_/MIL-88A_0.5_@Cit–gCN was determined using the XRD characterization tool (Fig. 1b). In the XRD pattern of pure gCN, two characteristic peaks were observed at 2*θ* values of 19.77° and 29.73°, associated with the planes of (100) and (002), respectively, according to the Joint Committee on Powder Diffraction Standards (JCPDS) no. 87-1526.^42^ The Cit–gCN pattern showed the same pattern as gCN, which may be due to the low Cit concentration in the analyzed sample.^43^ In the MIL-88A pattern, the corresponding peaks of MIL-88A emerged at 2*θ* of 10.77°, 11.98°, 12.94°, 15.37°, and 21.42°, belonging to the (100), (101), (110), (012), and (022) planes, respectively.^29^ In the FeS pattern, the diffraction peaks at 2*θ* of 25.57°, 30.06°, 36.40°, 47.88°, 52.41°, and 61.76° were related to the (111), (200), (211), (220), (311), and (023) planes from JCPDS no. 29-0723, respectively.^44^ In the FeS_0.5_/MIL-88A_0.5_@Cit–gCN pattern, a notable decline in the corresponding peaks of the pure components was observed, implying their bonding together.

#### 3.1.3. ZP

The charges on the surface of the FeS_0.5_/MIL-88A_0.5_@Cit–gCN catalyst were measured by the ZP technique, as presented in Fig. 1c. The ZP findings demonstrate that the average charges on FeS_0.5_/MIL-88A_0.5_@Cit–gCN at the pH values of 3, 5, 7, 9, and 11 are 7.22, 1.89, −11.29, −26.22, and −29.99 mV, respectively. It is concluded from the ZP curve that the FeS_0.5_/MIL-88A_0.5_@Cit–gCN catalyst has no charge at pH = 5.29. FeS_0.5_/MIL-88A_0.5_@Cit–gCN carries negative charges on its surface at a pH higher than 5.29; on the contrary, the surface charge of the catalyst becomes positive at a pH lower than 5.29.

#### 3.1.4. SEM

**Fig. 2:**
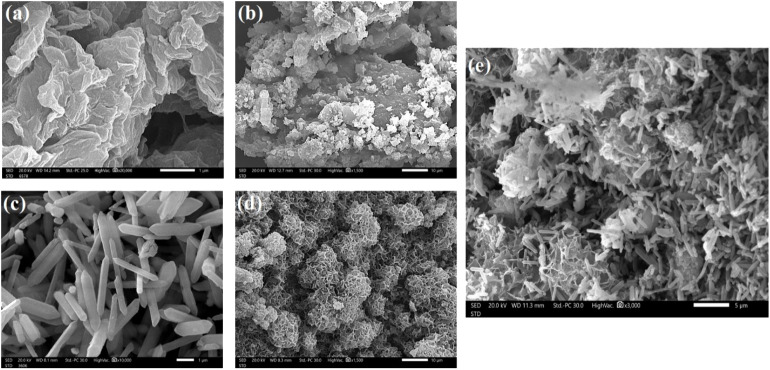
SEM images of (a) gCN, (b) Cit–gCN, (c) MIL-88A, (d) FeS, and (e) FeS_0.5_/MIL-88A_0.5_@Cit–gCN.

The outer morphologies of FeS, MIL-88A, gCN, Cit–gCN, and FeS_0.5_/MIL-88A_0.5_@Cit–gCN were scrutinized from the SEM images, as shown in Fig. 2a–e. For the gCN morphology, SEM elucidates the stacking of crumpled sheets with grooves and rough surfaces, endowing it with the characteristics of the excellent supporter. For the Cit–gCN morphology, the Cit particles are distributed on gCN sheets, confirming the Cit functionality, and this decoration likely increases the intersheet spacing and porosity, with an expected increase in its surface area. For the MIL-88A morphology, SEM reveals the common rod-like structure of the MIL-88A fabricated in aqueous media *via* the hydrothermal method. For the FeS morphology, SEM shows a flower-like morphology, which usually offers a large external surface and pore volume. For FeS_0.5_/MIL-88A_0.5_@Cit–gCN, the SEM image shows the uniform coverage of Cit–gCN sheets with the FeS and MIL-88A particles, which is expected to enhance the total pore volume and surface area. As a result, this combination is expected to provide a high surface area, which is the main factor in any efficient surface interaction.

#### 3.1.5. XPS

**Fig. 3:**
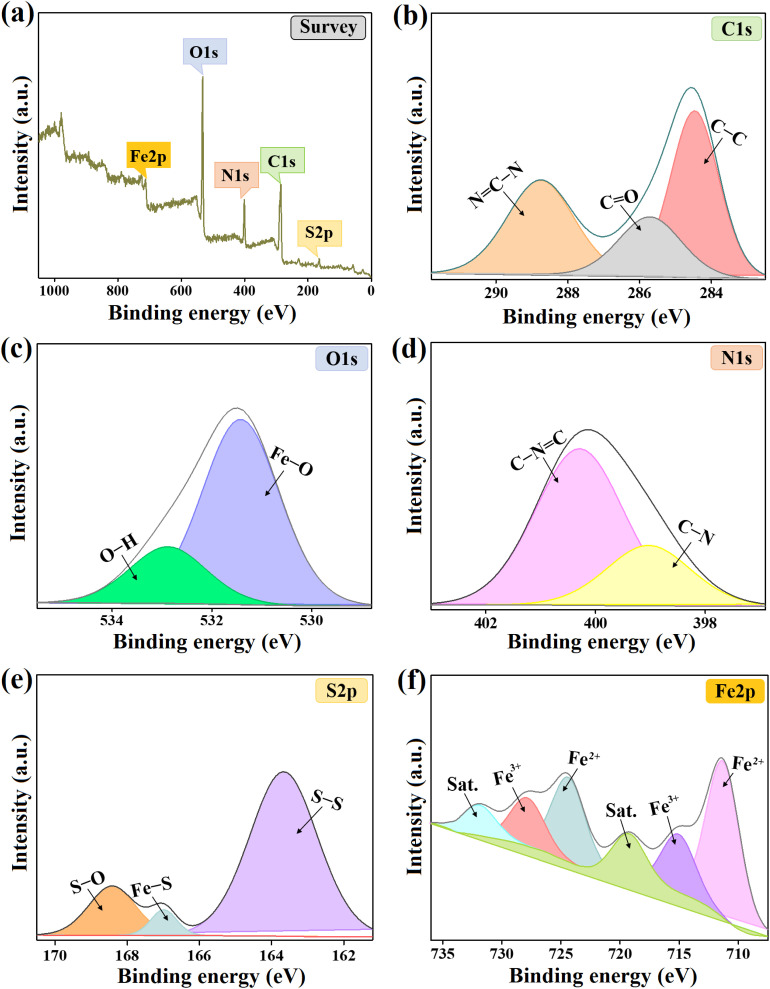
XPS spectra of pure FeS_0.5_/MIL-88A_0.5_@Cit–gCN: (a) wide spectrum and (b) C 1s, (c) O 1s, (d) N 1s, (e) S 2p, and (f) Fe 2p spectra.

The elemental composition of FeS_0.5_/MIL-88A_0.5_@Cit–gCN was investigated by the XPS tool, as illustrated in Fig. 3a–f. The XPS spectrum of FeS_0.5_/MIL-88A_0.5_@Cit–gCN presents the peaks belonging to carbon, nitrogen, oxygen, iron, and sulfur at 286.07, 400.93, 532.45, 712.45, and 164.83 eV, and their atomic percents in the composite are 53.23%, 12.81%, 27.97%, 2.94%, and 3.05%, respectively. In the carbon spectrum, the peaks corresponding to N=C–N, C–C, and C=O are revealed at 288.76, 284.47, and 285.71 eV, with atomic percents of 35.03%, 44.43%, and 20.53%, respectively. In the oxygen spectrum, the characteristic Fe–O peak (79.86%) of MIL-88A is observed at 531.34 eV, and the hydroxyl peak (20.14%) is observed at 533.11 eV. In the nitrogen spectrum, the peaks related to C–N=C and C–N are manifested at 400.29 and 399.03 eV, with atomic percents of 73.10% and 26.90%, respectively. In the sulfur spectrum, the Fe–S peak (4.74%), accompanying the iron sulfide, appears at 166.99 eV, and the peak at a binding energy of 168.42 eV represents the S–O bond (16.28%).^31^ In addition, the peak associated with the S–S bond emerges at 163.67 eV, with a total atomic percent of 78.98%.^45^ In the iron spectrum, the peaks at 715.08 and 727.85 eV signalize the ferric species, while the ferrous species peaks are manifested at 711.28 and 724.37 eV. Furthermore, the net atomic percents of ferric and ferrous species in the FeS_0.5_/MIL-88A_0.5_@Cit–gCN composites are 25.29% and 56.55%, respectively.

### 3.2. Optimization of the Fenton-like decomposition of Dox

#### 3.2.1. Optimizing the composite's ratio

**Fig. 4:**
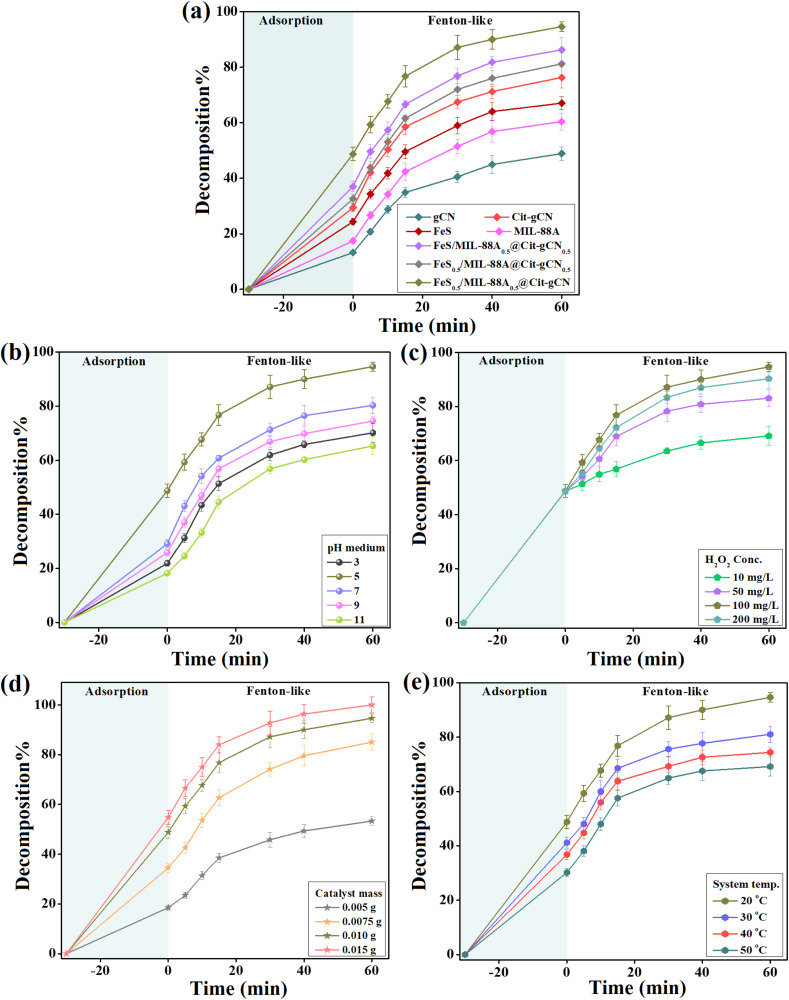
Experimental results of the adsorption/Fenton-like decomposition of Dox by the FeS_0.5_/MIL-88A_0.5_@Cit–gCN composite: (a) comparison between the catalytic activity of the composites and pure components toward decomposing Dox, and optimization tests showing the effect of the (b) pH medium, (c) H_2_O_2_ concentration, (d) FeS_0.5_/MIL-88A_0.5_@Cit–gCN dose, and (e) system temperature on the decomposition% of Dox.

A sequence of adsorption/Fenton-like experiments for decomposing the Dox molecules by gCN, Cit–gCN, FeS, MIL-88A, and FeS_0.5_/MIL-88A_0.5_@Cit–gCN was conducted to determine the improvement in the catalytic activity of gCN, as shown in Fig. 4a. The adsorption% and decomposition% of Dox by gCN were 13.28% and 48.92%, respectively, indicating its low catalytic activity, which requires enhancement. Surprisingly, the Cit functionality revealed an amelioration in the catalytic activity of gCN because the adsorption% and decomposition% of Dox reached 29.44% and 76.33%, respectively. This enhancement can be assigned to electron-rich groups in Cit that share their electrons for activating H_2_O_2_ and producing ˙OH. Furthermore, the Cit modification increased the gCN′ surface area, elevating its interaction with Dox. Moreover, the adsorption efficacies of FeS and MIL-88A toward Dox were 17.46% and 24.39%, and their decomposition percentages were 60.46% and 67.10%, respectively. Consequently, it was expected that binding FeS and MIL-88A with Cit–gCN could boost the composite's adsorption/catalytic activity by increasing its surface area as well as introducing active iron species and sulfur atoms from FeS. Likewise, MIL-88A has catalytic active groups like oxygenated functional groups and iron ions. These active species possess a high capacity for activating H_2_O_2_ and producing ˙OH. As a result, the FeS/MIL-88A@Cit–gCN composites exhibited an enhanced catalytic activity toward decomposing Dox compared to the pure components, where FeS_0.5_/MIL-88A_0.5_@Cit–gCN revealed a higher adsorption% of 48.77% and decomposition% of 94.66%, reflecting the synergistic effect between FeS, MIL-88A, and Cit–gCN.

#### 3.2.2. Optimizing the pH medium

The best pH medium to decompose Dox by FeS_0.5_/MIL-88A_0.5_@Cit–gCN with high efficiency was selected after testing the Fenton-like process at various pH values ranging from 3 to 11. The decomposition% *vs.* pH curve (Fig. 4b) signalizes the favorability of pH 5, where the adsorption% is 48.77%, and the Fenton-like decomposition% is 94.66%. Dox is one of the amphoteric drugs with a zwitter character at pH between 3.5 and 7.07, and it is a cation at pH < 3.5 and an anion at pH > 7.07. The FeS_0.5_/MIL-88A_0.5_@Cit–gCN composite has a neutral character at pH = 5.29, which increases the electrostatic interaction with the zwitter Dox. This observation endows merit to FeS_0.5_/MIL-88A_0.5_@Cit–gCN because the electrostatic repulsion is the obstacle to the adsorption process of such an amphoteric drug. Furthermore, the excess hydroxyl groups in neutral and basic media may be the cause of the declining Fenton-like decomposition of Dox at a pH higher than 5 because they interact with H_2_O_2_ and produce hydro-peroxy anions, which attack the metal ions in the Dox/FeS_0.5_/MIL-88A_0.5_@Cit–gCN system, as clarified in eqn (2) and (3). Additionally, the self-decomposition of H_2_O_2_ degrades it into water and carbon dioxide, dwindling the produced concentration of ˙OH radicals, as shown in eqn (4).

2 H_2_O_2_ + OH^−^ → OOH^−^ + H_2_O

3 M + OOH^−^ → M⋯˙OOH

4 2H_2_O_2_ → O_2_ + 2H_2_O

#### 3.2.3. Optimizing the H_2_O_2_ concentration

Fig. 4c displays the decomposition efficacy of Dox by FeS_0.5_/MIL-88A_0.5_@Cit–gCN in the presence of various concentrations of H_2_O_2_. It was recorded that raising the concentrations of the H_2_O_2_ molecules from 10 to 100 mg L^−1^ in the Dox-FeS_0.5_/MIL-88A_0.5_@Cit–gCN system boosted the decomposition% of Dox from 69.15% to 94.66%, but further increasing it to 200 mg L^−1^ declined the percent of decomposed Dox to 90.33%. This catalytic performance could be ascribed to the fostered production of the ˙OH radicals by the elevated concentration of the H_2_O_2_ oxidant. Furthermore, excess concentrations of H_2_O_2_ in the Dox-FeS_0.5_/MIL-88A_0.5_@Cit–gCN system could chelate the ˙OH radicals and create ˙OOH, as elucidated in eqn (5) and (6).

5 H_2_O_2_ + ˙OH → HOO˙ + H_2_O

6 HOO˙ + ˙OH → H_2_O + O_2_

#### 3.2.4. Optimizing the FeS_0.5_/MIL-88A_0.5_@Cit–gCN dosage

To determine the economical dosage to decompose the Dox molecules from wastewater with high efficiency, the FeS_0.5_/MIL-88A_0.5_@Cit–gCN dosage was optimized, as presented in Fig. 4d. It was recorded that the adsorption% of Dox dramatically improved from 18.50% to 48.77% by elevating the FeS_0.5_/MIL-88A_0.5_@Cit–gCN dosage from 0.005 g to 0.01 g, but further raising the dosage to 0.015 g caused an increase of only 6%. This result can be explained by the high concentrations of the provided active adsorption groups that chelate the Dox molecules from the polluted water. Likewise, the Dox decomposition% almost doubled when the FeS_0.5_/MIL-88A_0.5_@Cit–gCN dosage increased from 0.005 to 0.01 g, but further increasing the FeS_0.5_/MIL-88A_0.5_@Cit–gCN dose to 0.015 g did not reveal a significant effect. This catalytic behavior may be due to the enrichment of the system with highly active groups when the FeS_0.5_/MIL-88A_0.5_@Cit–gCN dosage is 0.01 g, which can produce higher amounts of ˙OH, decomposing higher concentrations of Dox molecules.^35^ Consequently, 0.01 g is the optimal FeS_0.5_/MIL-88A_0.5_@Cit–gCN dose, taking into consideration the economic point of view.

#### 3.2.5. Optimizing the system temperature

Fig. 4e displays the decomposition% of Dox by FeS_0.5_/MIL-88A_0.5_@Cit–gCN at increasing temperatures from room temperature to 50 °C. The adsorption% of Dox diminished from 48.77% to 30.18%; in addition, the Dox decomposition% declined from 94.66% to 69.1%. Such a decrease in the decomposition efficiency of Dox occurs with raising the system temperature since the Brownian motion of Dox inside the catalytic system becomes faster, diminishing the number of Dox molecules that reaches the FeS_0.5_/MIL-88A_0.5_@Cit–gCN surface.^46^

From the adsorption/Fenton-like optimization experiments, we deduced that the best mass ratio for FeS, MIL-88A, and Cit–gCN was 1 : 1 : 2. In addition, the optimal catalytic parameters for the decomposition process of Dox were system temperature = 20 °C, pH = 5, FeS_0.5_/MIL-88A_0.5_@Cit–gCN dose = 0.01 g, and H_2_O_2_ concentration = 100 mg L^−1^.

### 3.3. Kinetic study

**Fig. 5:**
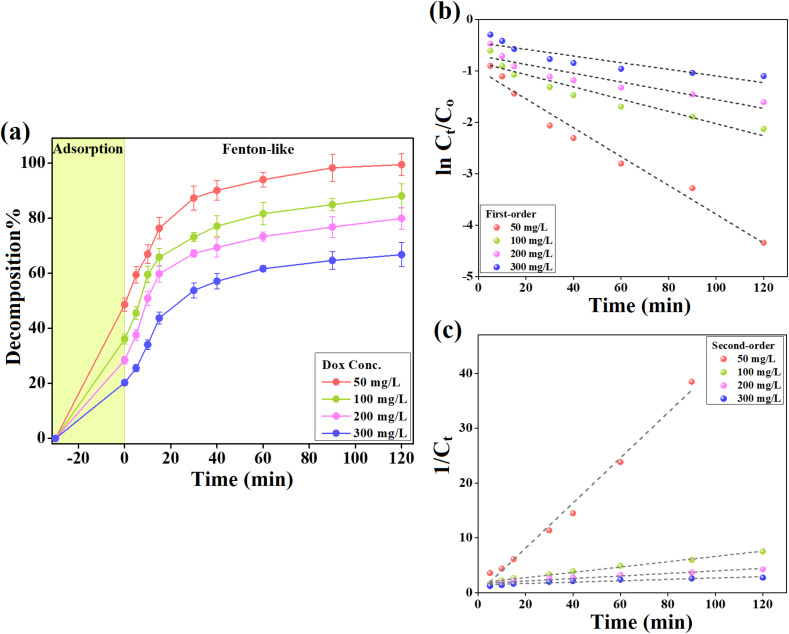
(a) Catalytic activity of FeS_0.5_/MIL-88A_0.5_@Cit–gCN toward decomposing various Dox concentrations, and kinetic analysis of the Fenton-like decomposition of Dox: (b) first-order and (c) second-order plots.

The catalytic activity of the Fenton-like FeS_0.5_/MIL-88A_0.5_@Cit–gCN catalyst was investigated toward Dox at concentrations between 50 and 300 mg L^−1^. As illustrated in Fig. 5a, the Dox adsorption% on the FeS_0.5_/MIL-88A_0.5_@Cit–gCN surface declined from 48.77% to 20.27% as the Dox concentration increased from 50 to 300 mg L^−1^. Meanwhile, the increase in the Dox concentrations diminished the decomposition% of Dox from 99.40% to 66.27%. These findings can be explained by the insufficient amount of active groups on the FeS_0.5_/MIL-88A_0.5_@Cit–gCN surface to adsorb and produce enough ˙OH to decompose the higher concentrations of Dox.^35^

The kinetics of the decomposition process of Dox by FeS_0.5_/MIL-88A_0.5_@Cit–gCN was inspected by the catalytic first-order and second-order models (eqn (7) and (8), respectively).7
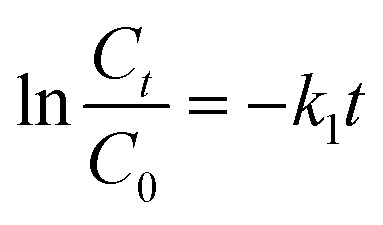
8
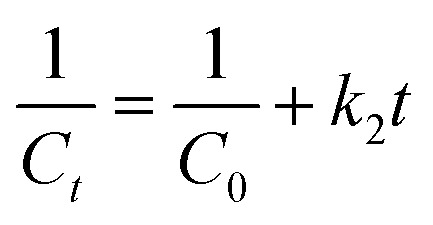
where *k*_1_ and *k*_2_ are the rate constants of the first-order and second-order models, respectively.

As revealed in Table 1, the decomposition reaction of Dox by FeS_0.5_/MIL-88A_0.5_@Cit–gCN obeyed the second-order kinetic expression, and the correlation coefficients of the linear second-order plot were higher than those of the first-order plot (Fig. 5b and c). The rate constants of the decomposition reaction of 50, 100, 200, and 300 mg L^−1^ Dox by FeS_0.5_/MIL-88A_0.5_@Cit–gCN decreased from 0.4260 to 0.0485, 0.0232, and 0.0127 L mol^−1^ min^−1^, respectively. This observation is most likely due to the complex interplay of catalytic parameters in the decomposition reaction, such as the simultaneous production and consumption of ˙OH radicals. Furthermore, compounds formed during the decomposition of Dox could compete with Dox molecules within the catalytic system.^47–49^

**Table 1:** Parameters derived from the kinetic study of the Fenton-like decomposition of Dox by FeS_0.5_/MIL-88A_0.5_@Cit–gCN

Kinetic model	Dox concentrations (mg L^−1^)			
	50	100	200	300
**First-order**				
*k*_1_	0.0281	0.0119	0.0086	0.0065
*R* ^2^	0.968	0.909	0.847	0.818

**Second-order**				
*k*_2_	0.4260	0.0485	0.0232	0.0127
*R* ^2^	0.992	0.990	0.946	0.889

### 3.4. Adsorption/Fenton-like mechanisms

**Fig. 6:**
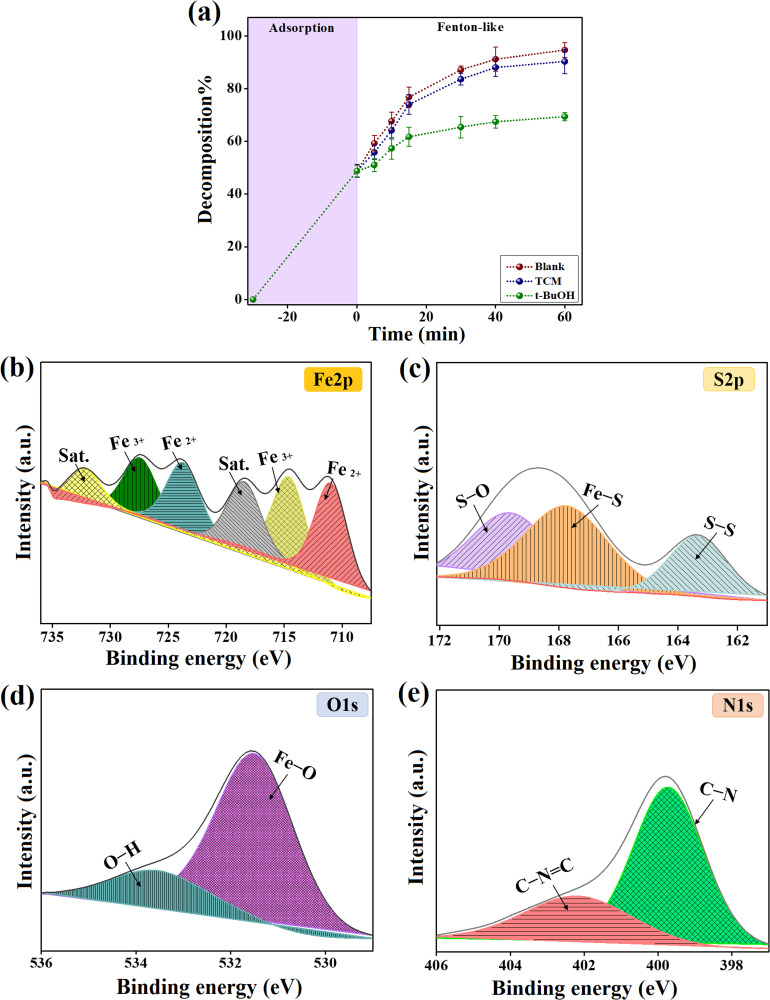
(a) Quenching test of the decomposition process of Dox by FeS_0.5_/MIL-88A_0.5_@Cit–gCN and XPS spectra of used FeS_0.5_/MIL-88A_0.5_@Cit–gCN: (b) Fe 2p, (c) S 2p, (d) O 1s, and (e) N 1s.

To determine the type of catalytic decomposition mechanism for Dox by FeS_0.5_/MIL-88A_0.5_@Cit–gCN, the scavenging test was performed in the presence of TCM and *t*-BuOH, separately, as illustrated in Fig. 6a. TCM and *t*-BuOH are the common scavengers used to explore the domination of the active O_2_˙− and ˙OH radicals, respectively, in the catalytic media owing to their ability to slow down the activity of these radicals.^50^ The experimental observations signalize the diminution in the decomposition capability of FeS_0.5_/MIL-88A_0.5_@Cit–gCN toward Dox, reaching 69.39%, in the presence of *t*-BuOH, implying that ˙OH is the active radical in the Dox-FeS_0.5_/MIL-88A_0.5_@Cit–gCN system.^51^ Consequently, the catalytic decomposition reaction of Dox by FeS_0.5_/MIL-88A_0.5_@Cit–gCN occurs throughout the radical pathway mechanism.

XPS spectra signalizes the production process of ˙OH in the Fenton-like catalytic Dox-FeS_0.5_/MIL-88A_0.5_@Cit–gCN system, as clarified below:

(i) The iron species are the typical active species in activating H_2_O_2_ and yielding ˙OH, following the catalytic Haber–Weiss process (eqn (9)). Notably, the iron species in FeS_0.5_/MIL-88A_0.5_@Cit–gCN are divided into 56.55% of Fe^2+^ and 25.29% of Fe^3+^ with a Fe^3+^/Fe^2+^ ratio of 0.447. By contrast, the XPS spectrum of the used FeS_0.5_/MIL-88A_0.5_@Cit–gCN reveals an elevation in the Fe^3+^/Fe^2+^ ratio to 0.852, suggesting the consumption of a part of Fe^2+^ during the Fenton-like decomposition of Dox in the production reaction of ˙OH (Fig. 6b). Moreover, the shifting in the iron peaks is another clue confirming its role in the Fenton-like decomposition of Dox; the Fe^2+^ peaks shift from 711.28 and 724.37 eV to 710.94 and 723.71 eV, and the position of the Fe^3+^ peaks changes from 715.08 and 727.85 eV to 714.57 and 727.44 eV, respectively.

(ii) The sulfur species in the FeS_0.5_/MIL-88A_0.5_@Cit–gCN catalyst can foster the catalytic activity of the near-iron species by presenting defects and transferring electrons from the Fe–S bond to the catalyst. Therefore, the unsaturated sulfur ions increase the shared electrons from FeS_0.5_/MIL-88A_0.5_@Cit–gCN to activate H_2_O_2_ and create ˙OH. The sulfur spectrum of the FeS_0.5_/MIL-88A_0.5_@Cit–gCN catalyst after the Dox decomposition (Fig. 6c) clarifies changes in the position of the sulfur peaks; Fe–S, S–O, and S–S peaks shift from 166.99, 168.42, and 163.67 eV to 168.40, 169.95, and 163.31 eV, respectively, proving its contribution in the H_2_O_2_ activation.

(iii) The FeS_0.5_/MIL-88A_0.5_@Cit–gCN catalyst possesses many electron-donating groups, like hydroxyl, tri-*s*-triazine, and carboxyl, which can participate in the Fenton-like decomposition of Dox by donating electrons to the catalytic medium for producing ˙OH, as presented in eqn (10). Moreover, such groups can regenerate the oxidized iron species to reuse them in the decomposition process of the Dox molecules (eqn (11)). Hence, the electron-donor groups of FeS_0.5_/MIL-88A_0.5_@Cit–gCN can initiate the decomposition process of Dox and maintain the continuity of the Fe^3+^/Fe^2+^ redox cycle. Fig. 6d displays a variation in the positions of the Fe–O and hydroxyl peaks of the used FeS_0.5_/MIL-88A_0.5_@Cit–gCN catalyst from 531.34 and 533.11 eV to 531.47 and 533.60 eV, respectively. Also, the peaks of the nitrogen-containing functional groups C–N=C and C–N shift from 400.29 and 399.03 eV to 402.20 and 399.72 eV, respectively, as illustrated in Fig. 6e. These observations ensure the involvement of the hydroxyl, tri-*s*-triazine, and carboxyl groups in the decomposition reaction of the Dox molecules.

Shortly, the iron, sulfur, hydroxyl, tri-*s*-triazine, and carboxyl species of FeS_0.5_/MIL-88A_0.5_@Cit–gCN activate H_2_O_2_ and produce ˙OH, which decomposes the Dox molecules to smaller compounds, resulting in the formation of CO_2_ and H_2_O, as clarified in eqn (12).

9 Fe^2+^ + H_2_O_2_ → Fe^3+^ + ˙OH + OH^−^

10 EDG + H_2_O_2_ → ˙OH + OH^−^

11 EDG + Fe^3+^ → Fe^2+^

12 Dox + ˙OH → intermediates → CO_2_ + H_2_O

Notably, the experimental results reveal the significant role of the adsorption reaction in the capacity of the Dox decomposition; so, it is important to suggest the mechanism of adsorbing Dox onto FeS_0.5_/MIL-88A_0.5_@Cit–gCN, as follows: (i) Dox can adsorb onto FeS_0.5_/MIL-88A_0.5_@Cit–gCN *via* the coulombic interaction between the zwitter Dox and the zwitter catalyst at pH ∼5. The presence of FeS_0.5_/MIL-88A_0.5_@Cit–gCN in a zwitter state at the same pH at which the amphoteric Dox exists in its zwitter character grants the catalyst a special merit because it overcomes the possible repulsion forces that are usually generated during the adsorption reaction of such amphoteric drugs and decline their adsorption capacity.

(ii) The iron species not only activate H_2_O_2_ to create ˙OH, but also play a worthy role during the adsorption reaction, where they can attach to the electron-donor groups of Dox (*viz.*, hydroxyl and amine) throughout coordination bonds.^52,53^ Furthermore, the presence of nitrogen and oxygen atoms in both FeS_0.5_/MIL-88A_0.5_@Cit–gCN and Dox suggests the H-bonding between the N and O of Dox and the H of the catalyst. In addition, there is a possibility of the N and O of FeS_0.5_/MIL-88A_0.5_@Cit–gCN chelating Dox by forming H-bonds with its H atoms.

(iii) n–pi interactions are a governing pathway in the adsorption reaction of Dox because its empty pi-orbital is available to accept electrons from the catalyst. The FeS_0.5_/MIL-88A_0.5_@Cit–gCN catalyst is rich with electron-donor active groups, comprising OH, tri-*s*-triazine, and COOH that share electrons with the empty pi-orbital of Dox; so, the catalyst can adsorb the Dox molecules onto its surface by forming n–pi interactions.^54^

### 3.5. Decomposition pathway

**Fig. 7:**
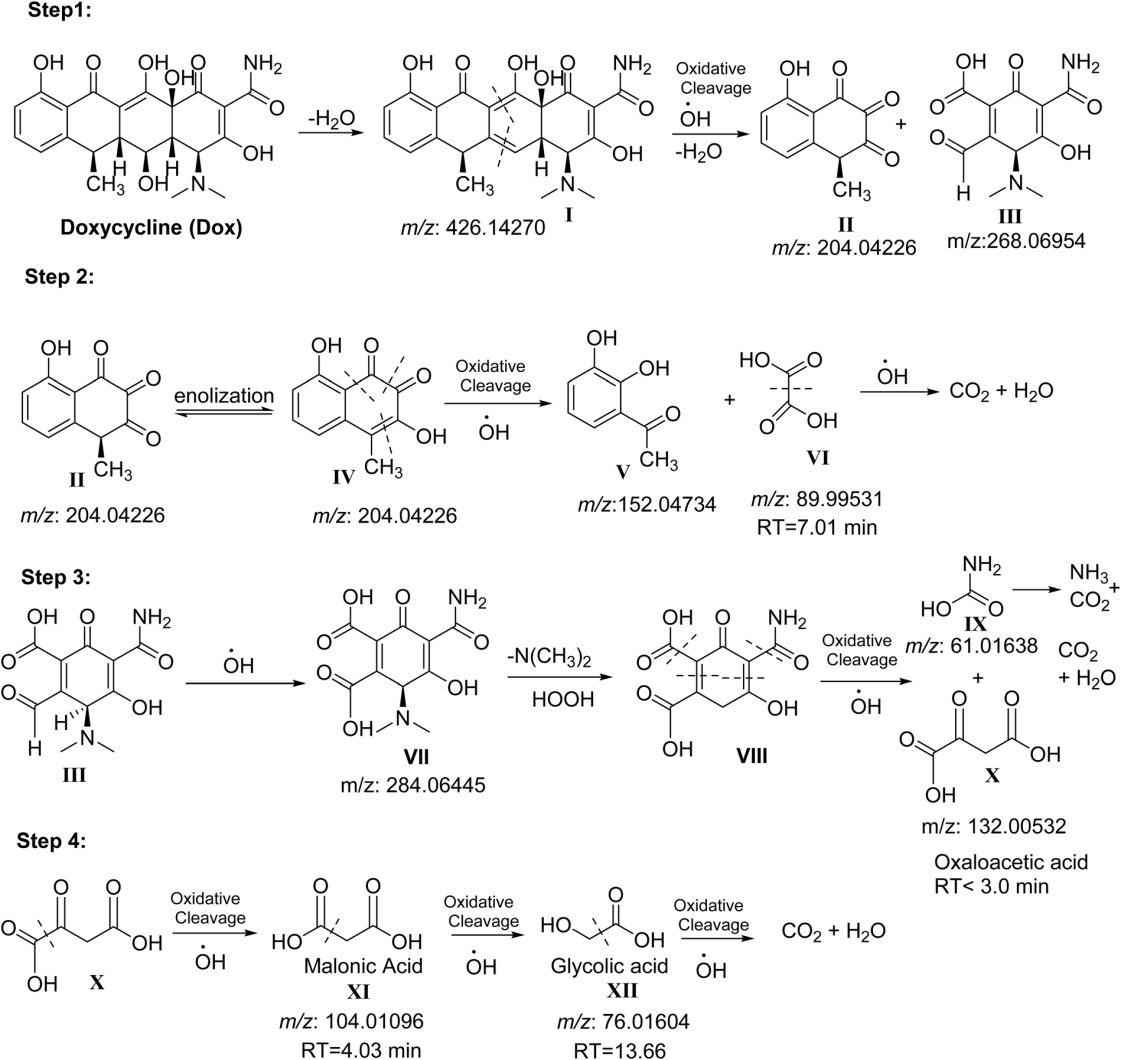
Schematic of the adsorption/Fenton-like decomposition of Dox by FeS_0.5_/MIL-88A_0.5_@Cit–gCN.

The degradation pathway of Dox by FeS_0.5_/MIL-88A_0.5_@Cit–gCN was suggested, based on the GC-MS and retention time results, as illustrated in Fig. S1. The degradation pathway includes 4 steps, as illustrated in Fig. 7. In the first step, Dox loses one molecule of water to afford the conjugated diene I with an experimentally identified mass of *m*/*z* = 425.3, which agrees with the calculated mass of [M − H] = 425.1, with I undergoing oxidative cleavage by ˙OH radicals to afford the corresponding key fragments (II and III). The degradation of the fragment (II) occurs in step 2 *via* enolization to afford IV, which decomposes by oxidative cleavage to afford acetyl catechol (V) and oxalic acid (VI). This pathway is confirmed by the result of GC-MS, where the RT of oxalic acid is 7.01, which agrees with the reported value in the literature and the mass of *m*/*z* = 91.2 corresponding to [M + 2H]. The decomposition of oxalic acid finally gives CO_2_ and water. The third step includes the degradation of the key compound, the reactive fragment III, which oxidizes rapidly to the corresponding acid (VII) and then loses dimethylamine, followed by hydrogen radical transfer to give (VIII). VIII is decomposed *via* oxidation to give the unstable carbamic acid (IX) and oxaloacetic acid (X). Carbamic acid degrades directly to ammonia and CO_2_. Oxaloacetic acid (X) has a low RT, less than 3.0 min, because its stability is limited. Experimental *m*/*z* of the oxaloacetic acid (X) = 134.5 which agree with the theoretical *m*/*z* value of the corresponding [M + 2H]. In the last step, the decarboxylation of X affords malonic acid XI with an RT of 4.03 min, which oxidizes to glycolic acid XII with RT = 13.66 min, agreeing with the reported value in the literature.^55,56^

### 3.6. Recycling investigation

**Fig. 8:**
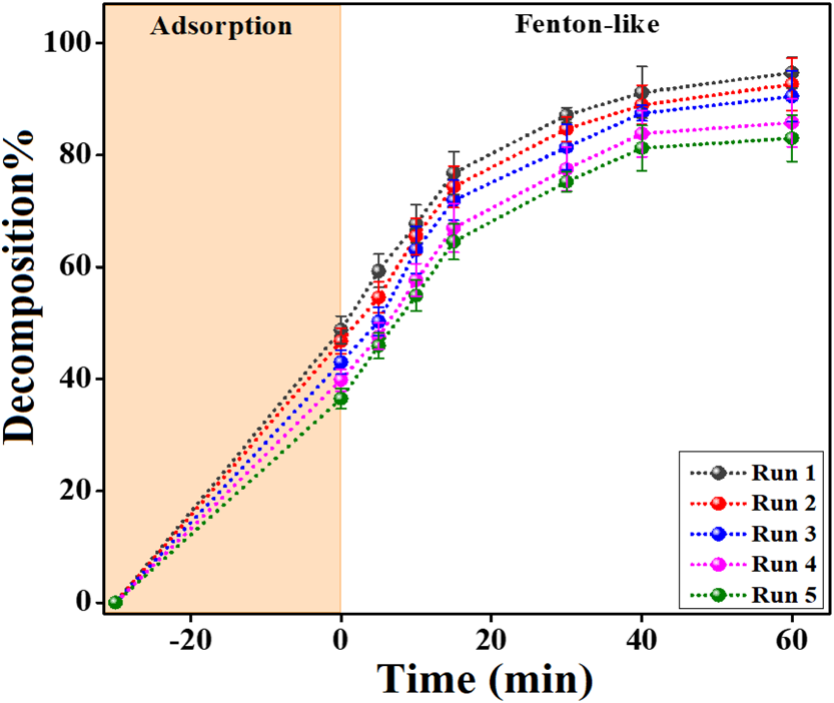
Cycling study of the FeS_0.5_/MIL-88A_0.5_@Cit–gCN catalyst during five decomposition runs of Dox.

The durability and reusability of the Fenton-like FeS_0.5_/MIL-88A_0.5_@Cit–gCN catalyst are essential criteria that must be met before recommending its use on an industrial scale. Therefore, the adsorption/Fenton-like cycling test was performed on the FeS_0.5_/MIL-88A_0.5_@Cit–gCN catalyst for five runs to investigate the capability of its recyclability feature. The cycling curve in Fig. 8 displays an insignificant decline in the adsorption% and decomposition% of Dox by FeS_0.5_/MIL-88A_0.5_@Cit–gCN by about 12.29% and 11.64%, respectively. Such a diminution in the adsorption% of Dox can be ascribed to the potent chemical and physical interactions between Dox and FeS_0.5_/MIL-88A_0.5_@Cit–gCN that hinder the complete desorption of the Dox particles during the recovery stage. Consequently, the adsorbed Dox on the active sites of FeS_0.5_/MIL-88A_0.5_@Cit–gCN shields those active groups and prevents them from donating electrons for activating H_2_O_2_. In addition, some mass of FeS_0.5_/MIL-88A_0.5_@Cit–gCN can be lost during the washing step of the catalyst recovery process.

## 4. Conclusion

The Fenton-like heterogeneous FeS_0.5_/MIL-88A_0.5_@Cit–gCN catalyst revealed an eminent catalytic activity towards decomposing Dox, where the adsorption% was 48.78% after half an hour and the decomposition% was 99.40% after two hours at pH = 5, temperature = 20 °C, and H_2_O_2_ concentration = 100 mg L^−1^. The zeta potential results implied that the zero charge point of FeS_0.5_/MIL-88A_0.5_@Cit–gCN was at pH = 5.29, endowing the catalyst an advantage because it overcame the electrostatic repulsion problem that hinders the adsorption of amphoteric Dox. Furthermore, the SEM of FeS_0.5_/MIL-88A0_.5_@Cit–gCN elucidated the presence of FeS and MIL-88A on the surface of Cit–gCN. The XPS spectra of used/genuine FeS_0.5_/MIL-88A_0.5_@Cit–gCN depicted the participation of its iron, sulfur, and electron-donor groups in activating H_2_O_2_ and creating ˙OH for decomposing Dox. In addition, the adsorption of Dox onto FeS_0.5_/MIL-88A_0.5_@Cit–gCN occurred through n–pi interactions, coulombic interactions, and coordination bonds. The recycling study showed a slight decrease in the activity during FeS_0.5_/MIL-88A_0.5_@Cit–gCN adsorption/Fenton-like decomposition cycles of Dox.

Future studies are recommended to expand the redox cycle by incorporating additional metal species through metal doping or by integrating metal-rich materials such as layered double hydroxides, MXenes, metal ferrites/oxides, or MOFs into the composite structure. Furthermore, the decline in the activity of FeS_0.5_/MIL-88A_0.5_@Cit–gCN during its recycling and reuse during Dox decomposition may be attributed to mass loss; so, adding magnetic materials to the composite or encapsulating it inside a polymeric matrix in a bead shape may be a suitable solution to this bottleneck. Moreover, a comprehensive study of composite surface characteristics will provide deeper insights into the surface-related phenomena governing catalytic performance.

## Conflicts of interest

There are no conflicts to declare.

## Funding

This work was supported by the Deanship of Scientific Research, Vice Presidency for Graduate Studies and Scientific Research, King Faisal University, Saudi Arabia [Project No. KFU253362].

## Supplementary Material

RA-015-D5RA07120H-s001

## Data Availability

The datasets supporting the findings of this study are available within the article and its supplementary information (SI) files. Supplementary information is available. See DOI: https://doi.org/10.1039/d5ra07120h.
