# Supplementary material for: Enhanced degradation of doxycycline by citric acid-functionalized graphitic carbon nitride decorated with MIL-88A and FeS: optimization, degradation mechanism, and degradation pathway
Source: RSC Adv. 2025 Dec 10;15(57):48994–9008. doi: 10.1039/d5ra07120h (PMC12690287; doi:10.1039/d5ra07120h)
Supplement: RA-015-D5RA07120H-s001 [file RA-015-D5RA07120H-s001.pdf]

Supplementary Information (SI) for RSC Advances.
This journal is © The Royal Society of Chemistry 2025

Text 1: The materials that were used in the preparation of the FeS/MIL-88A@Cit-gCN composite.

Thiourea ($SC(NH_2)_2$) and ferric chloride hexahydrate ($FeCl_3.6H_2O$) were brought from Rankem (India). t-butanol (t-BuOH), ethanol (absolute; 99%) and sodium hydroxide (NaOH) were provided from Aladdin Reagent Co., Ltd. (China). Trichloromethane (TCM), fumaric acid (Fu. acid), and ethylene glycol (EG) were purchased from Alpha Chemika (India).

Text S2: The characterization instruments in analyzing the FeS/MIL-88A@Cit-gCN composite.

The composition, morphology, and surface charge of the FeS/MIL-88A@Cit-gCN composite and its components were assessed by utilizing Zeta Potential (ZP., Malvern), X-Ray Diffraction (XRD., PANalytical), Fourier Transform Infrared (FTIR., Frontier, PerkinElmer), Scanning Electron Microscope (SEM., JEOL 7500F), and X-Ray Photoelectron Spectroscopy (XPS., ESCALAB 250XI). The produced compounds from decomposing the Dox molecules were deduced by Gas chromatography–mass spectrometry (GC-MS., SHIMADZU, GCMS-QP 2010 Ultra).

Figure S1. GC-MS of degradation product of Dox with $FeS_{0.5}$/MIL-88$A_{0.5}$@Cit-gCN.

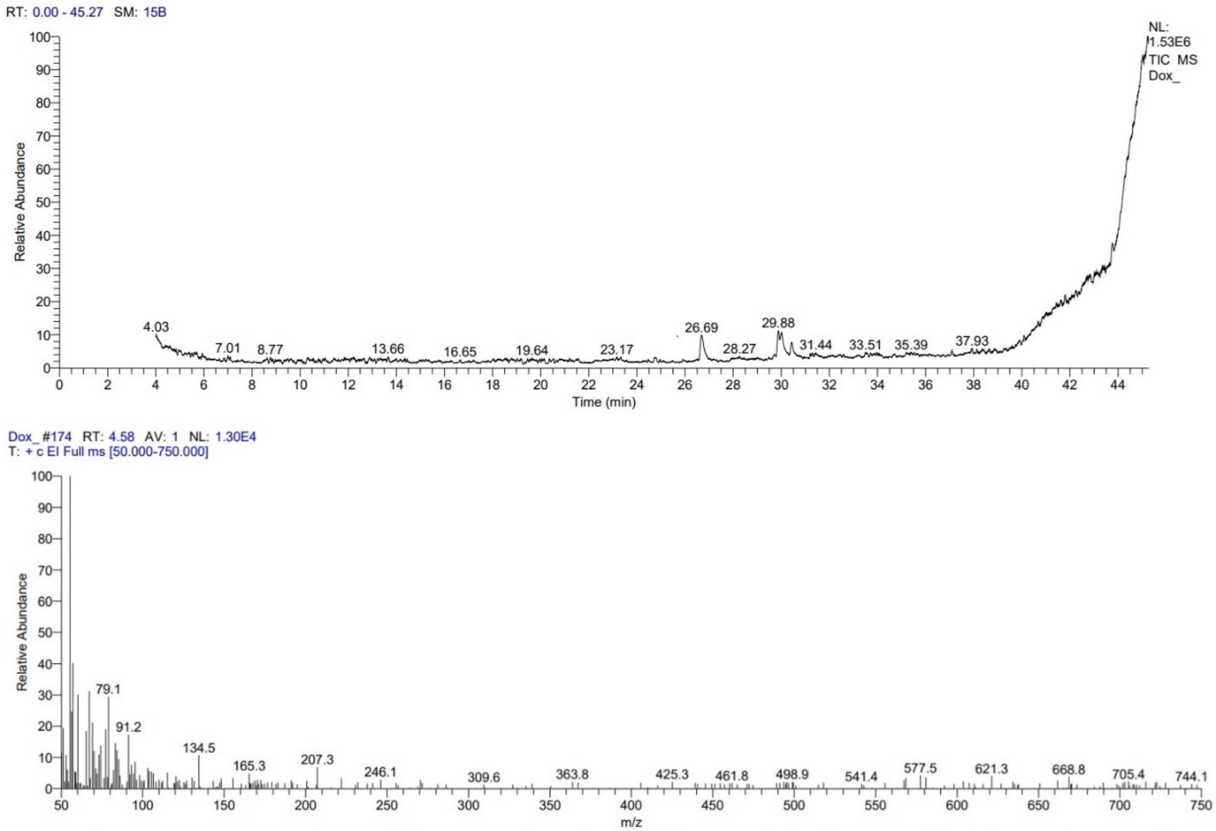

RT	Compound Name	Area %	MF	Molecular Formula	Molecular Weight	Cas #	Library
